# Getting ready for reduced donor dependency: the co-financing of family planning commodities

**DOI:** 10.1093/heapol/czad106

**Published:** 2023-11-07

**Authors:** Ayan Jha, Robert John Kolesar, Sophia Comas, Jay Gribble, Jorge Ugaz, Eduardo Gonzalez-Pier

**Affiliations:** Palladium Group, 1331 Pennsylvania Avenue NW, Suite 600, Washington, DC 20004, USA; Palladium Group, 1331 Pennsylvania Avenue NW, Suite 600, Washington, DC 20004, USA; Palladium Group, 1331 Pennsylvania Avenue NW, Suite 600, Washington, DC 20004, USA; Palladium Group, 1331 Pennsylvania Avenue NW, Suite 600, Washington, DC 20004, USA; Palladium Group, 1331 Pennsylvania Avenue NW, Suite 600, Washington, DC 20004, USA; Palladium Group, 1331 Pennsylvania Avenue NW, Suite 600, Washington, DC 20004, USA

**Keywords:** Health financing, donor policies, developing countries, family planning

## Abstract

Family planning (FP) programmes in low and lower-middle income countries are confronting the dual impact of reduced external donor commitments and stagnant or reduced domestic financing, worsened by economic consequences of the COVID-19 pandemic. Co-financing—a donor-government agreement to jointly fund aspects of a programme, with transition towards the government assuming increasing responsibility for total cost—can be a powerful tool to help build national ownership, fiscal sustainability and programme visibility. Using Gavi’s successful co-financing model as reference, the current paper draws out a set of key considerations for developing policies on co-financing of FP commodities in resource-poor settings. Macroeconomic and contextual sensitivities must be incorporated while classifying countries and determining co-financing obligations—using the actual GNI per capita on a scale or sovereign credit ratings, in conjunction with programmatic indicators, may be preferred. It is also important for policies to allow sufficiently long time for countries to transition—dependent on the country context, may be up to 10 years as allowed under the US Agency for International Development FP graduation policy and flexibility to revisit the terms following externalities that can influence the fiscal space for health. Incentivizing new domestic financing to pay for co-financing dues is critical, so as not to displace government funding from related health or social sector programs. Pragmatic ways to ensure country compliance can include engaging both the ministries of health and finance as co-signatories to identify and address known administrative and fiscal challenges; establishing dedicated co-financing account with the finance ministry; and instituting a mutual monitoring mechanism. Lastly, the overall process of policymaking can benefit from an alignment of goals and interests of the key development partners.

Key messages

- Co-financing policies can benefit from using sensitive economic measures reflecting countries’ ability to pay in conjunction with programmatic parameters; being flexible in modifying the terms and conditions should change economic externalities; and allowing for a sufficiently long period of time for countries (dependent on the country context, may be up to 10 years as allowed under the US Agency for International Development Family Planning graduation policy) to transition to self-financing.
- Engaging the Ministry of Finance along with the Ministry of Health early in the policymaking process is crucial to developing an inclusive approach that facilitates country compliance, stimulates and rewards new domestic financing to meet co-financing obligations, and thereby ensures fiscal sustainability.
- Key development partners should work to align their goals and interests to avoid fragmentation and redundancy of resources, establish uniform monitoring and reporting requirements, and possibly consider promoting co-financing under the larger umbrella of development assistance for primary health care, health system strengthening, health security and health financing reforms

## Introduction

The COVID-19 pandemic has contributed to a complex economic crisis reverberating around the world, and disproportionately affecting low- and lower-middle income countries (LICs, LMICs). In 2021, only 21% of LICs achieved their pre-pandemic level of economic productivity, compared to 40% of high-income counterparts. The situation is expected to worsen with a projected sharp decline in global growth in 2023, consequent to stringent fiscal policies adopted in response to unprecedented geopolitical strains, inflationary trends, and accumulating debt [United Nations Conference on Trade and Development (UNCTAD), 2021; World BanK, 2023]. The increased health spending observed during COVID-19 response was mostly accomplished through borrowing. Even without the stark predictions for 2023, it was projected that LICs may not recover to their pre-pandemic levels of government health spending until 2024 (Kurowski *et al.*, 2021).

This situation gives rise to unique challenges in sustainably financing family planning (FP) programmes in LICs and LMICs that are heavily dependent on external donor financing. Development assistance for FP accounted for 49% of total spending in 2018, but has plateaued over the last decade and near future commitments remain uncertain (Ali and Bellows, 2017). Further, private out-of-pocket expenditure has been the largest share of FP financing (Ali And Bellows, 2017)—amounting to estimated USD 2.73 billion in 2019 across 132 LICs and LMICs, with 90% being spent on contraceptive pills, condoms and injectables (Weinberger *et al.*, 2021).

The dual impact of reduced donor commitments and stagnant or reduced domestic financing is already disrupting FP programmes in some countries (Rigsby *et al.*, 2019). This equates to less access and choice, more unmet need, and reduced equity with a heavier financial burden on the poorest population in need of these essential health services. Innovative financing approaches that stimulate new domestic financing and hence ensure fiscal sustainability of FP programmes are needed to address this challenge. Indeed, national FP programmes have a proven track-record of being one of the most cost-effective primary health care interventions. The benefits of investing in FP programmes are realized not only through direct population health gains like improving maternal and neonatal mortality and morbidity, but also through increased female participation in the labour force, labour market productivity and macroeconomic effects such as a higher savings rate. Every dollar invested in addressing the unmet need for contraception can potentially save US$2.50 in health costs and contribute to a gain of US$120 per year through increased economic activities [Sundaram *et al.*, 2019, Copenhagen Consensus, Family Planning 2020 (FP2020)].

**Figure 1.**
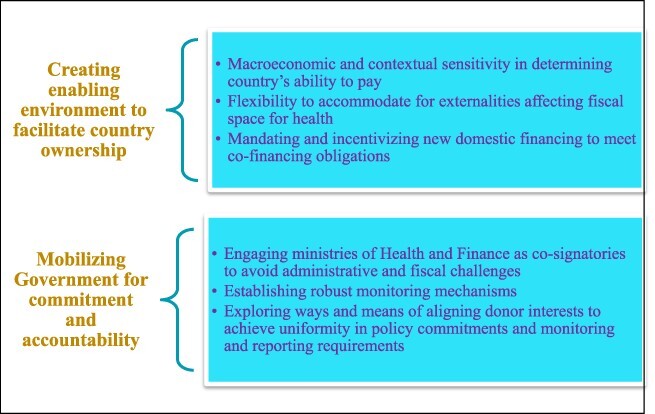
Overview of the key policy considerations for co-financing FP commodities

In this context, the current paper presents a set of key considerations (Figure 1) for developing policies on co-financing FP commodities in LICs and LMICs, based on work accomplished^1^ (Jha *et al.*, 2022) under the US Agency for International Development (USAID)-funded Health Policy Plus project. Co-financing refers to a donor-government agreement to jointly fund aspects of a programme, with transition towards the government assuming increasing responsibility for total cost. It has been successfully implemented by Gavi, the Vaccine Alliance to share fiscal responsibilities for introducing new and under-used vaccines ^2^ in LICs and LMICs (Gavi The Vaccine Alliance, 2016). Co-financing of FP commodities offers a fundamental re-alignment of external financing to facilitate structured transition from donor dependence, and is currently being considered by major donors (UNFPA, 2020; Jha *et al.*, 2022).

## Implementation

To develop the key considerations for FP co-financing policy, a mixed-method approach was implemented. Gavi’s co-financing model, which has a relatively long history and can be considered successful (Henderson *et al.*, 2016; Kolesar and Spruk, 2023), served as the reference. Annual progress reports were reviewed from 74 Gavi countries to identify common challenges; this was complemented with analysis of co-financing data from 64 FP2030 countries that received Gavi support between 2008 and 2019 (Gavi—The Vaccine Alliance, 2019; Family Planning 2020, 2022). In addition, a deeper dive into the experiences of Angola, Madagascar, and Nigeria was undertaken as illustrative case studies. Findings were translated to the FP policymaking context, cognizant that there may be less political will to increase domestic financing for FP programmes that are heavily donor reliant (Gotsadze *et al.*, 2019) as opposed to immunization programmes that enjoy more policymaking sensitivity.

## Challenges, enablers and constraints

### Creating an enabling environment to facilitate country ownership

The financial inability of a country to consistently fulfil its co-financing obligations was an important challenge identified in Gavi co-financing agreements. Based on the analyses, this can be attributed to the macroeconomic environment as rising inflation (adjusted Odds Ratio: 1.06; 90% confidence interval: 1.0003–1.13) and debt repayments (2.61; 1.27–5.36) significantly and negatively affected the ability to pay arrears. Further, inability to otherwise pay for annually escalating co-financing shares may have been associated with governments increasingly using external funding for their ‘traditional’ vaccination programme, suggesting a displacement of domestic with donor financing (Dieleman And Hanlon, 2014).

Nigeria, with government health expenditure (GHE) around 0.5% of GDP between 2010 and 2014, experienced displacement in its traditional vaccination programme over the same period despite a steady increase in the amount of government financing (Table 1). Nigeria’s co-financing share nearly doubled year-on-year from 2014 to 2016, when the country was reported not to have met its commitments. In a comparable example, Madagascar, with GHE 1.7–1.9% of GDP from 2010 to 2014, experienced displacement in its traditional vaccination programme over the same period (Table 2). While there is no evidence of a causal association between meeting co-financing dues and displacement, Madagascar was indeed unable to fulfill co-financing obligations from 2013 to 2018—a period over which its share increased by at least 30% year-on-year. In contrast, Angola did not experience any displacement in its traditional vaccination programme from 2010 to 2014 (Table 3). However, with its co-financing shares increasing more than 4-fold from 2012 to 2015, Angola was unable to meet its commitments from 2013 through 2017.

**Table 1.** Overall trends in financing the traditional vaccination programme, Nigeria (2010–2014)

	Select health financing indicators^a^				Total Govt. financing for traditional vaccination		Total donor financing for traditional vaccination	
Year	Domestic general government health expenditure (% of GDP)	Domestic general government health expenditure (% of general government expenditure)	Domestic general government health expenditure per capita (current US$)	Total expenditure, traditional vaccination programme, USD (Govt. + Donor)	USD	Total expenditure (%)	USD	Total expenditure (%)
2010	0.45	2.70	10.28	39 168 204	39 168 204	100	0	0
2011	0.48	2.77	12.00	112 620 977	39 076 739	34.7	73 544 238	65.3
2012	0.54	3.67	14.75	275 405 166	60 381 527	21.9	215 023 639	78.1
2013	0.49	3.46	14.42	230 497 083	216 373 220	93.9	14 123 863	6.1
2014	0.45	3.34	14.13	466 899 641	176 759 231	37.9	290 140 410	62.1

a **Source:** World Health Organization Global Health Expenditure database (http://apps.who.int/nha/database). The data were retrieved on 4 June 2023.
Domestic general government health expenditure (% of GDP): Public expenditure on health from domestic sources as a share of the economy as measured by GDP. Domestic general government health expenditure (% of general government expenditure): Public expenditure on health from domestic sources as a share of total public expenditure. It indicates the priority of the government to spend on health from own domestic public resources. Domestic general government health expenditure per capita (current US$): Public expenditure on health from domestic sources per capita expressed in current US dollars.

**Table 2.** Overall trends in financing the traditional vaccination programme, Madagascar (2010–2014)

	Select health financing indicators^a^				Total Govt. financing for traditional vaccination		Total donor financing for traditional vaccination	
Year	Domestic general government health expenditure (% of GDP)	Domestic general government health expenditure (% of general government expenditure)	Domestic general government health expenditure per capita (current US$)	Total expenditure, traditional vaccination programme, USD (Govt. + Donor)	USD	Total expenditure (%)	USD	Total expenditure (%)
2010	1.87	15.21	8.58	6 248 372	2 031 215	32.5	4 217 157	67.5
2011	1.68	13.94	8.67	1 608 502	429 983	26.7	1 178 519	73.3
2012	1.49	12.94	7.51	7 227 513	1 063 393	14.7	6 164 120	85.3
2013	1.30	10.27	6.87	7 656 331	1 241 771	16.2	6 414 560	83.8
2014	1.75	13.92	9.04	4 876 399	996 062	20.4	3 880 337	79.6

a **Source:** World Health Organization Global Health Expenditure database (http://apps.who.int/nha/database). The data were retrieved on 4 June 2023.
Domestic general government health expenditure (% of GDP): Public expenditure on health from domestic sources as a share of the economy as measured by GDP. Domestic general government health expenditure (% of general government expenditure): Public expenditure on health from domestic sources as a share of total public expenditure. It indicates the priority of the government to spend on health from own domestic public resources. Domestic general government health expenditure per capita (current US$): Public expenditure on health from domestic sources per capita expressed in current US dollars.

**Table 3.** Overall trends in financing the traditional vaccination programme, Angola (2010–2014)

	Select health financing indicators^a^				Total Govt. financing for traditional vaccination		Total donor financing for traditional vaccination	
Year	Domestic general government health expenditure (% of GDP)	Domestic general government health expenditure (% of general government expenditure)	Domestic general government health expenditure per capita (current US$)	Total expenditure, traditional vaccination programme, USD (Govt. + Donor)	USD	Total expenditure	USD	Total expenditure
2010	1.67	4.25	60.04	23 583 016	10 713 846	45.4	12 869 170	54.6
2011	1.71	4.58	78.95	37 700 403	24 988 489	66.3	12 711 914	33.7
2012	1.56	4.20	79.48	24 891 133	17 290 825	69.5	7 600 308	30.5
2013	1.70	4.58	88.63	39 835 391	37 758 886	94.8	2 076 505	5.2
2014	1.32	3.62	70.80	62 097 719	60 108 872	96.8	1 988 847	3.2

a **Source:** World Health Organization Global Health Expenditure database (http://apps.who.int/nha/database). The data were retrieved on 4 June 2023.
Domestic general government health expenditure (% of GDP): Public expenditure on health from domestic sources as a share of the economy as measured by GDP. Domestic general government health expenditure (% of general government expenditure): Public expenditure on health from domestic sources as a share of total public expenditure. It indicates the priority of the government to spend on health from own domestic public resources. Domestic general government health expenditure per capita (current US$): Public expenditure on health from domestic sources per capita expressed in current US dollars.

These case studies illustrate a common challenge: the need for more flexibility to accommodate evolving macroeconomic context in countries with limited domestic resources, which may contribute to enabling such countries fulfill their co-financing obligations, and the FP programme itself becoming more fiscally sustainable. As such, improved design of co-financing policies to account for this flexibility and generate new domestic spending might mitigate displacement of government funds from other health programmes Gavi uses the World Bank’s country income categories based on gross national income (GNI) per capita adjusted for annual inflation (Gavi the Vaccine Alliance, 2016) to determine countries’ ability to pay—but this approach may not account for instabilities in economic progress (Saxenian *et al.*, 2015) or incentivize new domestic spending to meet co-financing dues [Cambridge Economic Policy Associates (CEPA), 2019].

Hence, there is a need to accommodate more macroeconomic and contextual sensitivity while determining the basis for country classification, their initial co-financing shares, and subsequent rates of annual increase. Using the actual GNI per capita on a scale or sovereign credit ratings (The Global Economy.Com., 2022) in conjunction with programmatic indicators—similar to those outlined in USAID’s 2006 FP graduation policy (Chaudhry *et al.*, 2012; Jha *et al.*, 2022) has the potential to better assess countries’ ability to pay. Sovereign credit rating, unlike GNI, better reflects the government’s capacity to finance commitments. At the same time, it is important to gradually increase co-financing shares over a sufficiently long period of time (USAID FP graduation policy allows up to 10 years), and revisit the arrangement in annual joint review meetings following any major externalities that can influence the fiscal space for health. The recently launched UNFPA Supplies Partnership, which calls for minimum domestic financing contributions from participating countries (akin to co-financing), used GNI per capita, modern contraceptive prevalence rate (mCPR) and maternal mortality ratio (MMR) to determine eligibility of countries; while further using a complex economic index based on GNI per capita, World Bank income classification, and average GDP growth (except for LICs) to categorize selected countries based on ability to pay (UNFPA, 2020).

To ensure that FP co-financing does not lead to displacement of domestic government financing from related health or social sector programmes, agreements should both require and incentivize new government health spending, for example, through new tax revenues or spending efficiencies. Further, establishing a dedicated co-financing account with the Ministry of Finance (MOF) shall not only facilitate timeliness of payments, but also provide an opportunity to verify new domestic funding and increased budget transparency (Griffiths *et al.*, 2020). Besides monitoring conformity, national governments may indeed be rewarded for compliance—for example, by ensuring access to quality-assured commodities that meet regulatory standards (WHO-prequalified status) and are available at the best price for a substantially long period of time after transition to full domestic financing, in addition to continuing technical assistance to the national FP programmes.

### Mobilizing the government for commitment and accountability

The lack of coordination between Ministry of Health (MOH) and MOF was the most common reason for countries not meeting their co-financing commitments on time. MOF, which governs procedures of financial transactions including timing and modalities of paying dues, was not a co-signatory to Gavi co-financing agreements. Consequently, while the agreement required countries to pre-pay the vaccine supplier (UNICEF’s Supply Division) before delivery, this clause often contradicted MOF’s rules of post-delivery payment or at best, partial pre-delivery payment. In other instances, MOF’s fiscal year did not always align with Gavi, leading to administrative complexities that ultimately delayed payments to the vaccine supplier and led to digression from the co-financing agreement.

Typically, MOF oversees the multiyear budget plans and has a better understanding of national financial commitments to different development partners, including co-financing arrangements. In addition, as the MOF must balance myriad competing financing priorities, it is important that they are effectively engaged using compelling evidence as to the micro and macro-economic benefits of increasing financing for primary health care. However, health sector co-financing agreements often do not sufficiently engage MOF along with MOH—a fact noted not only with Gavi, but also with the 2019 Kenya FP commodities memorandum of understanding (MOU) between Kenya MOH, Gates Foundation, UK Foreign, Commonwealth and Development Office and USAID (Abonyo *et al.*, 2022).

Based on these findings, it seems pragmatic to engage both MOH and MOF at early stages of the discussion on co-financing agreements, so that administrative and fiscal challenges can be identified, and remedial measures initiated to avoid non-/delayed payments at later instances. Unless both these ministries become co-signatories, it is difficult to ensure full country commitment and fiscal sustainability of the venture. Notably, the ‘country compact’ under UNFPA Supplies Partnership mandates both MOH and MOF as co-signatories for co-financing of FP commodities (UNFPA, 2020).

Further, there is a need to put a robust monitoring mechanism in place with dedicated focal points to ensure action on commitments and communicate foreseeable challenges. Both the UNFPA compact, and Kenya MOU stipulate documentation of financial transactions as well as procurement, storage, and last mile distribution of FP commodities (UNFPA, 2020), but a dedicated structure to monitor commitments was still not in place in Kenya by mid-2022 (Abonyo *et al.*, 2022)—highlighting a critical operationalization gap.

Finally, possible alignment of the goals and interests of key development partners may simplify and facilitate country compliance with FP co-financing policies, since diverse approaches and reporting requirements have been noted as obstacles in implementing health programmes across LICs and LMICs (OECD, 2003). The current progress in developing FP co-financing policies presents an opportunity for donors and other stakeholders like Reproductive Health Supplies Coalition [Reproductive Health Supplies Coalition (RHSC), 2022] and ‘Shaping Equitable Market Access for Reproductive Health’ Partnership (Sema Reproductive Health, 2022) to synchronize their efforts. Such alignment can have major benefits through donors becoming sensitized on the cumulative effect of simultaneous co-financing commitments from different vertical programmes on the fiscal space for health; avoiding fragmentation and redundancy of external resources; and creation of uniform monitoring and reporting requirements for FP co-financing. While acknowledging that internal mandates and regulations can be a hindrance to inter-donor agency coordination, developing a financial sustainability plan for the entire spectra of donor financing to the health sector will not only be an efficient approach but also incentivize country buy-in and compliance.

## Conclusion

In the context of uncertain economic recovery from COVID-19, LICs and LMICs need to be strategic and committed in finding sustainable domestic resources to finance health—so that the hard-earned gains are not lost, and progress continues in expanding population coverage, service coverage, and financial risk protection. Financing and sustaining FP programmes in these circumstances presents very unique challenges—inertia of recipient LICs/ LMICs to evolve to self-financing from a traditionally donor-funded programme, the stagnated and likely declining level of external financing, and lack of political sensitivity when FP programmes must compete for financing with other essential health services. While co-financing is an attractive proposition for donors wanting to transition out of a country, the paper presents a set of key macro-level considerations which seek to strengthen the evidence-based gaps in policymaking.

It remains imperative to recognize that transitioning to full domestic financing in FP will take substantial time. Achieving this goal requires an inclusive process that focuses on the ways and means to incentivize countries to follow that path. To that end, integrating FP into the package of essential health benefits provided through national/sub-national universal health coverage (UHC) programmes/insurance schemes might offer a holistic option—as the MOH and MOF are more likely to take a favourable view of financing a UHC package of service offerings which is both equitable and efficient; and FP programmes stand to benefit from the system-wide efforts to strengthen public financial management, supply chain and human resources for health (Kutzin *et al.*, 2018). Development partners can certainly leverage this perspective, and consciously promote co-financing of FP commodities under the larger umbrella of development assistance for primary health care, health system strengthening, health security, and health financing reforms.

## Footnotes

1. The detailed report based on this work is publicly available at: http://www.healthpolicyplus.com/pubs.cfm?get=18658.

2. Only new and under-used vaccines are part of Gavi co-financing mechanism. Other ‘traditional’ vaccines like BCG, DPT, OPV/ IPV, MMR, TT, etc. are typically financed by government and various donors, including Gavi, but not linked to the co-financing mechanism.

## Why all named authors are from high-income countries:

This paper is based on work accomplished under the USAID-funded Health Policy Plus (HP+) project, which aimed to strengthen and advance health policy priorities, including those related to family planning, at global, national and subnational levels—exclusively focusing on low- and lower-middle-income countries in Africa, Asia, Latina America and the Caribbean. While HP+ worked through many in-country teams, the specific activity on which this paper is based (principles for co-financing of family planning commodities) was led by experts based in Palladium’s Washington, DC office. The paper is informed by the project’s combined experience working on different family planning activities spanning many countries. Among the authors, A.J. has lived and worked in India, R.J.K. has lived and worked in Cambodia, J.G. has lived and worked in Pakistan and E.G.P. has lived and worked in Mexico.

## Reflexivity Statement

The USAID-funded Health Policy Plus project, under which this work was accomplished, collaborated extensively with ministries of health across Africa, Asia, Latin American, and the Caribbean. The paper reflects the combined experience of the authors working with country teams and health leaders from resource-poor settings. It has benefitted from diverse technical perspectives: health financing expertise was provided by R.K.J., S.C., J.U. and E.G.P.; family planning expertise was provided by J.G.; and clinical and health systems expertise was provided by A.J. Within the authors’ group, S.C. is an early-career public health professional, A.J. is at the mid-senior level, and R.J.K., J.U., J.G. and E.G.P. are senior experts in their fields. Further, A.J., R.J.K. and J.G. have lived and worked as public health professionals in low/ lower-middle-income countries.
